# Resting-state functional MRI reveals altered brain connectivity and its correlation with motor dysfunction in a mouse model of Huntington’s disease

**DOI:** 10.1038/s41598-017-17026-5

**Published:** 2017-12-01

**Authors:** Qiang Li, Gang Li, Dan Wu, Hanbing Lu, Zhipeng Hou, Christopher A. Ross, Yihong Yang, Jiangyang Zhang, Wenzhen Duan

**Affiliations:** 1 Department of Radiology, Johns Hopkins University School of Medicine, Baltimore, MD USA; 2 Department of Radiology, Tangdu Hospital, Xi’an, Shaanxi China; 3 Division of Neurobiology, Department of Psychiatry and Behavioral Sciences, Johns Hopkins University School of Medicine, 600 N. Wolfe Street, Baltimore, MD 21287 USA; 4 Department of Pharmacology, Inner Mongolian Medical University School of Pharmacy, Inner Mongolian, China; 5 Intramural Research Program, National Institute on Drug Abuse, Baltimore, MD USA; 6 Department of Neuroscience, Johns Hopkins University School of Medicine, Baltimore, MD USA; 7 Department of Pharmacology and Molecular Sciences, Johns Hopkins University School of Medicine, Baltimore, MD USA; 8 Department of Radiology, New York University School of Medicine, New York, NY USA; 9 Program in Cellular and Molecular Medicine, Johns Hopkins University School of Medicine, Baltimore, MD USA

## Abstract

Huntington’s disease (HD) is an autosomal dominant inherited neurodegenerative disorder, and no cure is available currently. Treatment of HD is likely to be most beneficial in the early, possibly pre-manifestation stage. The challenge is to determine the best time for intervention and evaluate putative efficacy in the absence of clinical symptoms. Resting-state functional MRI may represent a promising tool to develop biomarker reflecting early neuronal dysfunction in HD brain, because it can examine multiple brain networks without confounding effects of cognitive ability, which makes the resting-state fMRI promising as a translational bridge between preclinical study in animal models and clinical findings in HD patients. In this study, we examined brain regional connectivity and its correlation to brain atrophy, as well as motor function in the 18-week-old N171-82Q HD mice. HD mice exhibited significantly altered functional connectivity in multiple networks. Particularly, the weaker intra-striatum connectivity was positively correlated with striatal atrophy, while striatum-retrosplenial cortex connectivity is negatively correlated with striatal atrophy. The resting-state brain regional connectivity had no significant correlation with motor deficits in HD mice. Our results suggest that altered brain connectivity detected by resting-state fMRI might serve as an early disease biomarker in HD.

## Introduction

Huntington disease (HD) is a neurodegenerative disorder resulting from a trinucleotide repeat expansion in the *huntingtin* gene. To date, proven neuroprotective strategies remain elusive. Part of the problem has been that most of the trials have attempted intervening at a time when the degenerative process is already far advanced and hence when it would be difficult even for the most effective therapy to demonstrate any benefit. Treatment of HD is likely to be most beneficial in the early, possibly pre-manifestation stage. The challenge is to determine the best time for intervention and how to evaluate putative neuroprotection in the absence of clinical symptoms. Therefore noninvasive and objective early biomarkers which are sensitive to changes in neuronal dysfunction during the presymptomatic phase are strongly needed.

Multiple neuroimaging modalities have been explored in this regard. Among the biomarkers, the striatal volume is a notably robust marker in HD patients reported by many studies^1–7^ as well as in HD mouse models in our previous studies^8–10^. Longitudinal studies have shown that brain structural volumes in manifest HD can track disease-related changes over time. However, atrophy of the striatum or cortex does not characterize the functional topography of disease progression^11,12^. Measurements of brain connectivity can add the dimension of brain function and may be sensitive to early functional changes in HD. In this regard, such measurements are of keen interest to elucidate the effect of early degenerative changes on intrinsic activity within the brain.

Brain activity at resting state exhibits intrinsic low frequency synchronization between anatomically distinct brain regions, a stimulus-free fMRI approach, defined as resting-state fMRI (rs-fMRI) has been developed during last decade. Rs-fMRI allows making temporal correlations between brain areas, based on spontaneous task-independent fluctuations of the blood-oxygenation level dependent (BOLD) signal in the brain^13,14^. It has been shown that altered functional brain connectivity detected by rs-fMRI in prodromal and early manifest HD subjects^15–21^, and a recent study demonstrated that an abnormal functional connectivity of the default mode network precedes brain atrophy and is sensitive to a drug treatment, may indicate a biomarker of dysfunction in HD^21^.

Research into development in biomarkers and therapies for HD is particularly attractive because it is a genetically homogeneous disease for which numerous well-established animal models exist. To date, however, no study has examined the resting-state functional networks in HD mouse models yet, and it is unknown whether the functional connectivity changes are correlated with brain pathology and behavioral phenotypes in HD mice. In this study, we investigated the functional connectivity by rs-fMRI and analyzed the relationship between functional connectivity and striatal atrophy, as well as motor function in a well-characterized HD mouse model- N171-82Q^9,22^.

## Results

### Independent components and resting-state functional networks are consistently identified in both control and HD mouse brains

**Figure 1:**
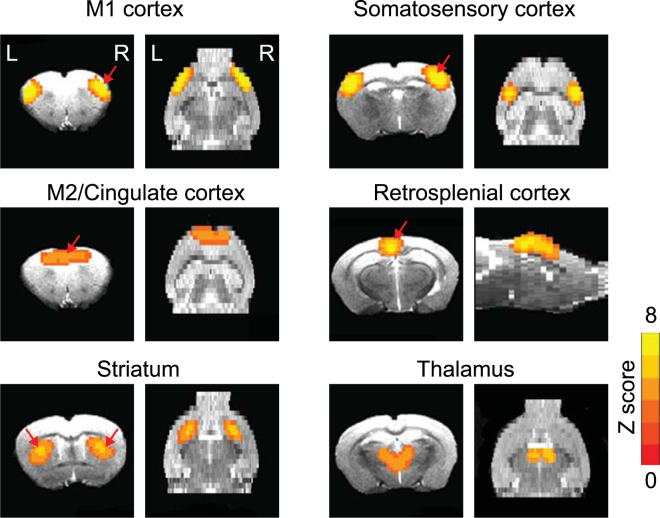
The key networks identified by rs-fMRI are detected in both control and HD mice include the primary motor cortex M1 network, secondary motor cortex M2/ cingulate cortex network, striatum network, somatosensory network, retrosplenial cortex network and thalamus network. Z Score from 0 to 8 as indicted by the color bar. The red arrows indicate the seed locations for the subsequent seed-based analysis.

To mitigate the inhibitory effects of the isoflurane on BOLD signals, the anesthesia regime used in this study combined dexdomitor (dexmedetomidine hydrochloride, 0.5 mg/mL, Pfizer Inc. NY, NY, USA) and low dose isoflurane ( < 0.5%). Using data-driven spatial independent component analysis (ICA), we identified six stable functional components, including the primary motor (M1) cortex, secondary motor (M2) and cingulate cortex, striatum, somatosensory cortex, retrosplenial cortex, thalamus networks (Fig. 1). These networks were consistently detected in both control and HD mouse brains, aligned well with anatomical boundaries, and were comparable with the networks reported in previous rs-fMRI studies of rodent models^23–26^.

### Quantitative inter-group comparison of functional connectivity between control and HD mouse brain reveals an HD specific pattern

**Figure 2:**
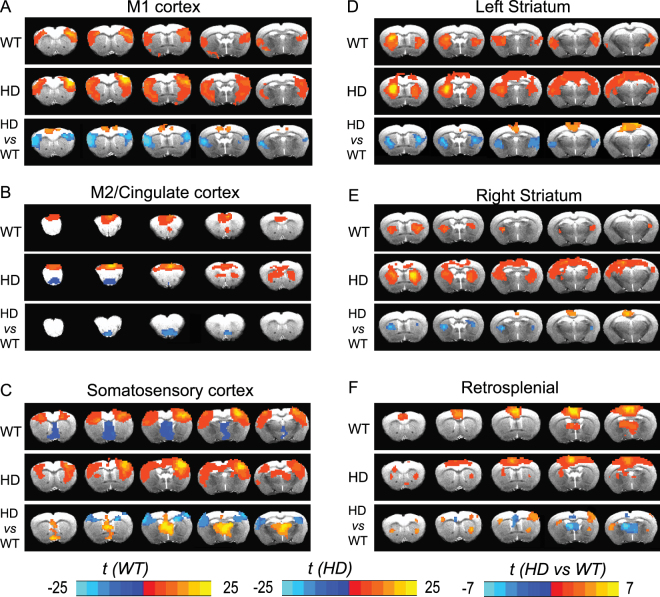
Significantly altered functional connectivity maps in N171–82Q HD mice versus their littermate controls. The seed locations in different brain regions were indicated in each panel. the blue color covered regions in the HD vs WT panels indicate the brain regions which have reduced connectivity with indicated seed regions in each panel, and orange covered regions indicate the regions which have enhanced connectivity with the seed regions. *n* = 7 (control group) and 13 (HD group), the value is HD/control. *t* values were indicated by the color bars (bottom of the graph), the range is from −25 to 25 in WT or HD panels, and from −7 to 7 in HD versus HD panels. p < 0.005, corrected with Monte Carlo simulation program.

In order to determine whether mutant huntingtin alters brain connectivity, we employed seed-based analysis in the networks, the seed located was indicated in in Fig. 1 (red arrows), and we compared the connectivity maps between the HD mice and age-matched controls. Significant perturbations in brain functional connectivity were evidenced in multiple networks in HD mice comparing with age-matched controls at 18 weeks of age (Fig. 2A–F). Specifically, the blue color covered regions in the HD vs WT panels indicate the brain regions which have reduced connectivity with indicated seed regions in each panel, and orange covered regions indicate the regions which have enhanced connectivity with the seed regions. We observed that the connectivity between bilateral M1 motor cortex regions was decreased in HD mice compared that in WT mice (Fig. 2A bottom panel). In addition, the connectivity between bilateral somatosensory cortex regions was decreased in HD mice (Fig. 2C); both intra- and inter- striatum connectivity were significantly decrease while the connectivity between striatum and retrosplenial cortex was enhanced in HD mice (Fig. 2D,E). Finally, the connectivity between retrosplenial cortex and thalamus was significantly reduced in HD mice (Fig. 2F).

### Correlations between striatal atrophy and functional connectivity in the HD mouse brain

**Figure 3:**
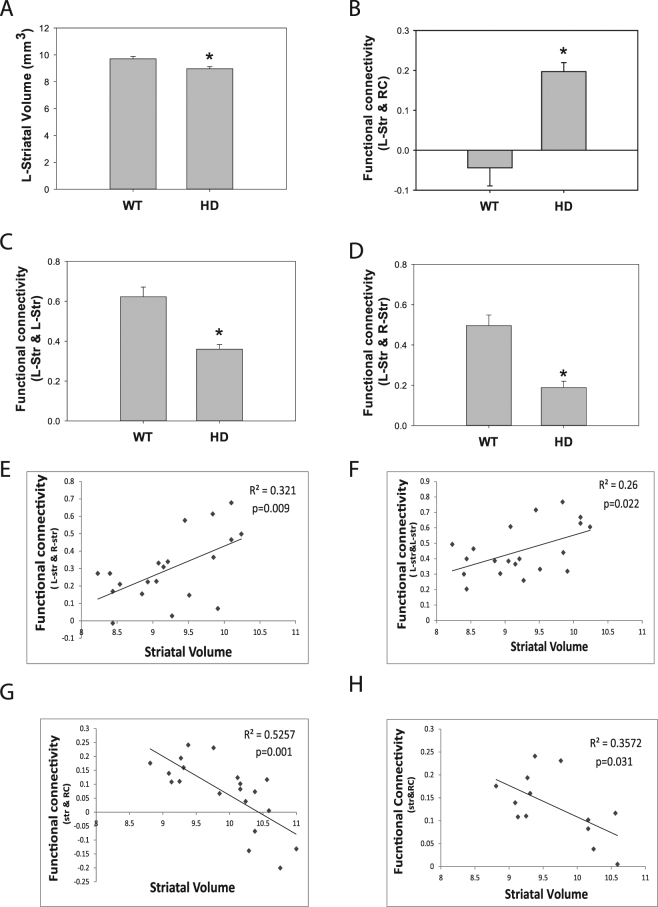
Correlation analysis between striatal volumes and resting-state brain regional functional connectivity in the N171–82Q HD (*n* = 13 mice) and their littermate control mice (*n* = 7 mice). (**A**–**D**) Striatal atrophy and altered resting-state functional connectivity was detected in HD mice compared those in age-match control mice. *p* < 0.05 compared to the value in the control group by standard Student’s *t*-test. (**E**) Positive correlation between striatal volume and bilateral striatum connectivity in all mice, *n* = 20. (**F**) Positive correlation between striatal volume and intra-striatal resting-state functional connectivity in all mice, *n* = 20. (**G**) Negative correlation between striatum volume and connectivity between striatum and retrosplenial cortex (RC) in all mice, *n* = 20. (**H**) Correlation between striatal volume and striatum-RC connectivity in HD mice only. *n* = 13. The correlation was analyzed by Pearsonian correlation modeling.

As previous demonstrated, N171–82Q HD mice exhibited striatal atrophy at 18 weeks of age (Fig. 3A). Significantly enhanced resting-state functional connectivity was detected between left striatum and retrosplenial cortex (RC) at the same age (Fig. 3B), whereas significantly reduced functional connectivity within left striatum was evidenced in the same cohort at the same age (Fig. 3C). Further correlation analysis indicated positive correlations between striatal atrophy and decreased inter-striatum functional connectivity (Fig. 3E, *p* = 0.009) and intra-striatum connectivity (Fig. 3F, *p* = 0.022), while a negative correlation was evidenced between reduced striatal volume and functional connectivity between retrosplenial cortex (RC) and striatum (Fig. 3G, *p* = 0.001) in all mice includeing wild type and HD mice (*n* = 20). Within the HD group, a significant negative correlation between striatal volume and functional connectivity between RC and striatum was reported (Fig. 3H, *p* = 0.031, *n* = 13).

### Correlations between motor function and functional connectivity in HD mice

**Figure 4:**
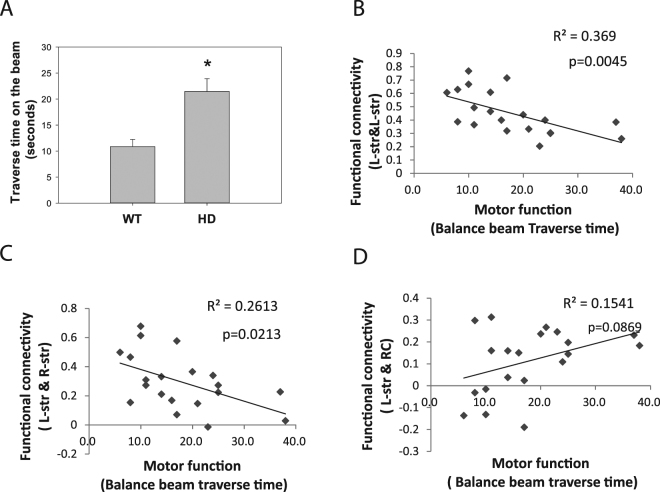
Correlation analysis between motor function and resting-state functional connectivity in the N171–82Q HD (*n* = 13) and their litter mate control (*n* = 7) mice. *n* = 20. (**A**) Motor deficits were evidenced in HD mice indicated by prolonged traverse time on the beam. *p* < 0.05 compared to the value in the control group by standard Student’s *t*-test. (**B**–**D**) Correlation between motor dysfunction and altered resting-state functional connectivity between brain regions indicated in the graphs was analyzed by Pearson correlation modeling. Retrosplenial cortex (RC), left striatum (L-str).

Next we determined whether the brain functional connectivity is correlated with motor function in HD mice, since motor deficits are more consistent phenotypes between HD mouse models and HD subjects. The motor function was assessed on a 5 mm balance beam in the same cohort of mice which we conducted fMRI scans. 18-week-old N171–82Q HD mice exhibited motor deficits indicated by extended traverse time on the beam (Fig. 4A). There was a significant positive correlation between motor performance and reduced intra-striatal functional connectivity (Fig. 4B, *p* = 0.0045) and inter-striatal functional connectivity (Fig. 4C, *p* = 0.0213), whereas the correlation between motor deficits and striatum-RC connectivity was not statistically significant (*p* = 0.0869) (Fig. 4D) in all mice (*n* = 20). Within the HD group, no significant correlation between motor function and functional connectivity measurements was identified. The R^2^ value between motor deficits and intra-striatal functional connectivity was 0.25 (*p* = 0.077, graph not shown).

## Discussion

Our results revealed, for the first time, perturbations in resting-state brain functional connectivity in a HD mouse model. Notably, we observed a robust association between altered striatal connectivity and striatal atrophy as well as motor performance. In this study we investigated rs-fMRI signals among functionally connected brain regions and used two complementary analysis approaches, independent components analysis showed that stable network interconnectivity in mouse brain, and seed-based analysis revealed patterns of weakened striatal connections and strengthened striatal to the retrosplenial cortex connections in the HD mice. These disturbances in functional connectivity were strongly associated with selective brain atrophy and motor performance, demonstrating that regional brain connectivity measures may provide a unique window into the structural and functional consequence induced by mutant huntingtin in the brain. These results may bring additional insights into the connections between human and mouse models of HD, e.g., how regionally specific neuronal dysfunction relates to specific phenotype and selective neuropathology.

Striatal atrophy is a neuropathological hallmark of HD even during the pre-manifest stage^3,4,27^. We explored what these structural changes mean for neural activity at rest. Statistical analysis of both WT and HD mice revealed that the weakened intra- and inter-striatal connectivity is positively correlated with striatal atrophy, while the strengthened striatal-retrosplenial cortex connectivity is negatively correlated with striatal atrophy. However, within the HD groups, we only detected a significant correlation between striatal volume and striatal-retrosplenial cortex connectivity. Where the results suggest potential connection between structural and functional measurements, there are additional factors involved in modulating the functional connectivity measurements. For example, it is unknown whether weakened functional connectivity reflects limited or disappeared networks due to neuronal death, which may be detected in structural measurements, or neuronal dysfunction, which may not be reflected in structural measurements. Future studies by combining fMRI and neuropathology in a longitudinal setting are warranted.

The impaired motor function is a consistent phenotype between human HD and HD mouse models; we therefore examined the association between altered functional connectivity with motor performance. Although there is correlation between motor performance and intra-striatal connectivity in all mice, but we the correlation within the HD group did not show statistical significance, thought there is a similar trend in HD mice (*n* = 13) versus all mice (*n* = 20). As motor behavioral measurements often show large variations, future studies with a larger number of HD mice than the current study may yield more definite results on the relationship between motor behavioral performance and functional measurements in HD mice.

It has been reported that HD patients showed connectivity changes within distinct resting state networks, including lateral prefrontal, supplementary motor, thalamic, cingulate, temporal and parietal regions, in HD patients, aberrant connectivity of resting-state networks associated with motor function^15–17,19,20^. Brain regions that show reduced intrinsic functional connectivity are present even in premanifest HD gene carriers and to a much larger extent in manifest HD patients^18^, therefore rs-fMRI could potentially be used for early disease detection in the pre-manifest phase of HD and for monitoring of disease modifying compounds in both preclinical and clinical studies in this population.

The strongest potential confounding factor when performing functional connectivity analysis in animals is the impact of anesthetics. Our previous studies in rats have used analgesic sedative, such as dexmedetomidine^24^, to limit the sedation level of the animals. However, maintaining a stable dose of these anesthetics for prolonged experiments (2 h) is challenging. More recently, it has been demonstrated that robust functional connectivity measures in mice could be consistently detected using optimized anesthesia regime by combination of low doses isoflurane and medetomidine^28–30^. Compared with other anesthetic regimes, the combination of low doses of isoflurane and medetomidine preserves resting-state networks within cortical and subcortical structures in mice, with similar results to those obtained in awake animals^31^. Therefore we combined low dose isoflurane and medetomidine in our current study, and detected consistent functional network in mouse brain.

Limitation of this study is the cross-sectional design, further longitudinal studies are needed to reveal dynamic changes of functional connectivity, and determine how the connectivity changes contribute to neuropathology as well as behavioral deficits in HD. The longitudinal studies will also be able to assess modifications of the pattern of the network alterations throughout the disease course, understand its role in the disease progression, and further clarify the mechanisms underlying these network alterations. Such studies will provide more insights into the exact relation between functional connectivity and brain pathology as well as clinical symptoms.

## Methods

### Animals

Transgenic N171–82Q HD mice were maintained by breeding heterozygous N171–82Q male mice with C3B6F1 female mice (Jackson Laboratory, Bar Harbor, ME, USA). DNA was obtained from tails of the offspring for determination of the genotype as we described previously^9^. The mice were housed in groups of 3–5 with access to food and water ad libitum and a 12-h reversed light/dark cycle. Male N171–82Q HD mice were used in this study (the HD group, *n* = 13), and their littermate control mice were used in this study as control (the WT group, *n* = 7). All experimental procedures were approved by the Animal Use and Care Committee at the Johns Hopkins University School of Medicine. All methods were performed in accordance with relevant guidelines and regulations.

### Imaging acquisition

*In vivo* MRI scans were performed on a horizontal 11.7 Tesla MR scanner (Bruker Biospin, Billerica, MA, USA) with a triple-axis gradient (maximum gradient strength = 740 mT/m). The scanner was also equipped with a physiological monitoring system (EKG, respiration, and body temperature, SAII instrument, Stony Brook, NY, USA). Body temperature and respiration rate were monitored via a rectal probe and pressure-sensitive respiratory sensor. Body temperature was maintained at 36.1 ± 1.4 °C throughout the imaging session by circulating warm water in the animal holder and warm air flow over the mice.

Images were acquired using a quadrature volume excitation coil (72 mm diameter) and a receive-only planar surface coil (15 mm diameter) placed on the top of the mouse head. Mice were initially anesthetized with isoflurane (1.5%) together with air and oxygen mixed at a 3:1 ratio via vaporizer during preparation. To mitigate the inhibitory effects of the isoflurane on BOLD signals, the anesthesia was immediately switched to combined dexdomitor (dexmedetomidine hydrochloride, 0.5 mg/mL, Pfizer Inc. NY, NY, USA) with low dose isoflurane (0–0.5%)^29,32^. Moderate dexdomitor sedation was initially induced by a bolus injection via i.p. (0.01 mg/kg body weight). The mice were then placed in an animal holder with ear pins and a bite bar to restrain head motions. The mice continued to receive air and oxygen mixed at a 3:1 ratio together with low dose isoflurane (0.5%). Ten minutes after the initial bolus injection, the mice started to receive a continuous subcutaneous infusion of dexdomitor through a MRI compatible catheter controlled by an infusion pump (Harvard Apparatus, Holliston, MA, USA) at a rate of 0.03 mg/kg body weight per hour. During imaging, the dose of isoflurane was manually adjusted between 0~0.5% to maintain a relatively stable respiratory rate (90–120 per minute).

Structural MR images were acquired using a two-dimensional multi-slice T2-weighted fast spin echo sequence, with the following parameters: echo time (TE)/repetition time (TR) = 40/3000 ms, field of view (FOV) = 20 × 20 mm^2^, matrix size = 192 × 192, in-plane resolution = 0.104 mm × 0.104 mm, 50 slices with a slice thickness of 0.3 mm and no gap, echo train length = 4, and 2 signal averages. At approximately 30 to 45 minutes after the initial bolus injection of dexdomitor, the fMRI data were acquired using gradient-echo echo-planer imaging (GE-EPI) with the following parameters: TE/TR = 13/1200 ms, flip angle = 60°, FOV = 20 × 20 mm^2^, matrix size = 80 × 80, in-plane resolution = 0.25 × 0.25 mm^2^, 21 slices with a slice thickness of 0.5 mm and no gap, number of volumes = 300, and 10 dummy scans. The fMRI acquisition was repeated for three times for each mouse. The mice recovered quickly once the anesthesia was turned off.

## Structure imaging analysis

### Initial processing

T2-weighted MR images were first rigidly aligned to a template image by using the automated image registration software (AIR, http://bishopw.loni.ucla.edu/AIR5/). The template image was selected from one of the images acquired from age-matched littermate control mice, which had been manually adjusted to the orientation defined by the Paxino’s atlas with an isotropic resolution of 0.1 × 0.1 × 0.1 mm^3^ per pixel. After rigid alignment, all images had the same position and orientation as the template image, and image resolution was also adjusted to an isotropic resolution of 0.1 × 0.1 × 0.1 mm^3^ per pixel. Signals from non-brain tissue were removed manually (skull-stripping).

### Computational analysis

Skull-stripped, rigidly aligned T2-weighted MR images were analyzed using the Diffeomap software (www.mristudio.org). Intensity values of the gray matter, white matter, and cerebral spinal fluid were normalized to the values in our MRI-based mouse brain atlas^8,33^ by using a piece-wise linear function. This procedure ensured that subject image and atlas image have similar intensity histograms. The intensity normalized images were submitted by the Diffeomap software to a linux cluster, which runs Large Deformation Diffeomorphic Metric Mapping (LDDMM). The atlas consists of an *in vivo* population averaged T2-weighted mouse brain image and segmentations of major gray and white matter structures^33^. The transformations deformed the structural segmentations in the atlas to the subject images to achieve automated segmentation of the subject images. The results of the automated segmentation were inspected and modified manually when necessary, and the volumes of each segmented structure were obtained. We have previously examined the accuracy of the computational analysis in another mouse models of HD^8^.

### fMRI data analysis

The fMRI data from individual animals were registered to a common space derived from registration of all the mice and subjected to the following preprocessed pipeline using the AFNI software package^34^: slice timing correction, linear and quadratic trend removal, band-pass (0.01–0.1 Hz) filtering, and spatial smoothing (0.6 mm FWHM). The average signal time course from the ventricles was removed from gray matter signal through linear regression.

### Independent component analysis

Group independent component analysis (ICA) was performed using GIFT v2.0a (http://www.nitrc.org/projects/gift/) in order to identify prominent functional brain networks. First, a two-step data reduction was conducted by principal component analysis (PCA), in which the fMRI data were reduced, followed by concatenation of the fMRI data in groups. Second, a group ICA was performed on the de-meaned data using the Infomax algorithm, with a brain mask to remove non-brain signals. Third, a back reconstruction of the data to individual subject independent components was also performed to examine the reproducibility of the components detected. The number of independent components (ICs) used for the analysis was tested in a pilot study of wild-type mice performed with varying component numbers (6, 10, 12, 15, 20). The number of IC selected (N = 15) was found to ensure maximal integrity and bi-laterality of fMRI signals in major gray matter structures without resulting in loss of spatial information^35^. All the other default parameters of GIFT were used. Independent components were scaled to z scores and thresholded at |Z| > 2, (corresponding to p < 0.05, two-tailed test). Components were then overlaid onto a co-registered T2-weighted MR image.

### Functional connectivity

Because motor-function-related networks are of interest in HD mice, we selected specific seed regions for the functional connectivity analysis from striatum, M1, M2/cingulate cortex, retrospenial cortex, somatosensory cortex, and thalamus networks identified in the ICA results. The seed regions included bilateral striatum, anterior cingulate cortex, M2 cortex, right M1 region and somatosensory cortex (minimum voxels = 20). A cross-correlation coefficient map for each seed region was obtained by correlating the average time course from the seed regions with the time course of each voxel within the brain. Fisher Z transformation was used to account for bounded values. The individual correlation maps from three scans were averaged to improve the functional connectivity (FC) maps. The averaged FC data for each mouse were treated as “one sample” in the statistical analysis.

### Behavioral tests

Mice were randomly divided into groups, the same animals which were scanned by MRI were used for motor function tests. Motor function was assessed on an 80-cm long and 5-mm wide square-shaped or 11-mm diameter round-shaped balance beam that is mounted on supports of 50-cm in height. A bright light illuminated the start platform, and a darkened enclosed 1728 cm^3^ escape box (12 × 12 × 12 cm) was situated at the end of the beam. Disposable pads were placed under the beam provided cushioning if an animal fell off the beam. Mice were trained to walk across the beam twice at least 1 h prior to testing. After the training trial, mice were left undisturbed for at least an hour before testing. The time for each mouse to traverse the balance beam was recorded with a 125-seconds maximum cut-off, and falls will be scored as 125 seconds.

### Statistics

Voxel-wise one-sample *t* tests (*P* < 0 0.001, minimum voxels = 20) were employed to obtain the functional connectivity maps of the each seed region in the two groups. Moreover, two-sample *t* tests (*P* < 0.005, minimum voxels = 20, based on Monte Carlo simulations^34^) were performed to compare functional connectivity of each seed region between two groups. The amplitude of functional connectivity from the differential regions between two groups was extracted. Correlations between regional functional connectivity and brain regional atrophy or motor behavioral performance were modeled using Pearson correlation analysis. Pearson coefficients were calculated from the slope. The significance was set at *P* < 0.05.

### Data availability

The datasets generated during and/or analyzed during the current study are available from the corresponding author on reasonable request.
